# Intrinsic functional connectivity predicts remission on antidepressants: a randomized controlled trial to identify clinically applicable imaging biomarkers

**DOI:** 10.1038/s41398-018-0100-3

**Published:** 2018-03-06

**Authors:** Andrea N. Goldstein-Piekarski, Brooke R. Staveland, Tali M. Ball, Jerome Yesavage, Mayuresh S. Korgaonkar, Leanne M. Williams

**Affiliations:** 1 Department of Psychiatry and Behavioral Sciences, Stanford University, 401 Quarry Road, Stanford, CA 94305 USA; 2 Sierra-Pacific Mental Illness Research, Education, and Clinical Center (MIRECC) Veterans Affairs Palo Alto Health Care System, 3801 Miranda Avenue, Palo Alto, CA 94304 USA; 3 Brain Dynamics Center, The Westmead Institute for Medical Research, University of Sydney, Sydney, NSW Australia; 4 Discipline of Psychiatry, Sydney Medical School, Westmead, Sydney, NSW Australia

## Abstract

Default mode network (DMN) dysfunction (particularly within the anterior cingulate cortex (ACC) and medial prefrontal cortex (mPFC)) has been implicated in major depressive disorder (MDD); however, its contribution to treatment outcome has not been clearly established. Here we tested the role of DMN functional connectivity as a general and differential biomarker for predicting treatment outcomes in a large, unmedicated adult sample with MDD. Seventy-five MDD outpatients completed fMRI scans before and 8 weeks after randomization to escitalopram, sertraline, or venlafaxine-XR. A whole-brain voxel-wise *t*-test identified profiles of pretreatment intrinsic functional connectivity that distinguished patients who were subsequently classified as remitters or non-remitters at follow-up. Connectivity was seeded in the PCC, an important node of the DMN. We further characterized differences between remitters, non-remitters, and 31 healthy controls and characterized changes pretreatment to posttreatment. Remitters were distinguished from non-remitters by relatively intact connectivity between the PCC and ACC/mPFC, not distinguishable from healthy controls, while non-remitters showed relative hypo-connectivity. In validation analyses, we demonstrate that PCC–ACC/mPFC connectivity predicts remission status with >80% cross-validated accuracy. In analyses testing whether intrinsic connectivity differentially relates to outcomes for a specific type of antidepressant, interaction models did not survive the corrected threshold. Our findings demonstrate that the overall capacity to remit on commonly used antidepressants may depend on intact organization of intrinsic functional connectivity between PCC and ACC/mPFC prior to treatment. The findings highlight the potential utility of functional scans for advancing a more precise approach to tailoring antidepressant treatment choices.

## Introduction

Major depressive disorder (MDD) is highly prevalent^1^ and a diagnosis of MDD alone does not inform us about which treatment choices will work best for the individual patient. As little as a third of patients may remit following the first choice of antidepressant^2,3^, and depression is now one of the leading causes of disability as well as a primary risk for suicide^4^. To address this burgeoning issue, as a field we are searching for markers that are predictive of remission and that have translational relevance to the clinic. A promising avenue for guiding classification and treatment choices is the development of a brain-based taxonomy for depression and related experiences^5,6^. Guided by this approach, in this study we focus on the intrinsic functional connectivity of the default mode network (DMN). Functional connectivity, particularly within the DMN is implicated in the pathophysiology of MDD^7–9^ and may have a role in differentiating responders from non-responders to treatments, including antidepressants^10–14^.

Previous studies have laid important foundations for the role of the DMN in depression, particularly the posterior cingulate cortex (PCC) and the medial prefrontal cortex (mPFC) nodes. The most consistent findings have identified aberrant functioning^15,16^ as well as connectivity both within and between these DMN nodes^11,15–19^, which may resolve after treatment^11,15^. Recently, two unique subtypes of depression, differentiated by the presence or absence of PCC–anterior cingulate cortex (ACC)/mPFC connectivity, were identified using community detection algorithms on resting-state functional data within a depressed sample^20^. This is particularly interesting when considering that differences in PCC–ACC/mPFC connectivity prior to treatment have been linked to treatment responsiveness in late-life depression^10^. While these past reports implicate DMN functional connectivity in the pathophysiology of depression and depression treatment response, we do not yet know the role of DMN intrinsic functional connectivity as a general and differential biomarker for predicting treatment outcomes in broader age range of depressed individuals. Further, no study to date has characterized the accuracy, sensitivity, and specificity of using intrinsic functional connectivity of the DMN as a prospective predictor of antidepressant outcomes.

Addressing these issues, in the current study we assessed which aspects of pretreatment dysfunction in the DMN predict antidepressant treatment outcomes more generally, and which aspects differentially predict outcome for different antidepressants. We investigated these issues in a patient sample from the International Study to Predict Optimize Treatment Outcomes for Depression (iSPOT-D), who were unmedicated at the pretreatment baseline and subsequently randomized to different antidepressants^21^. We hypothesized that pretreatment connectivity (particularly within the ACC/mPFC) with the PCC node of the DMN would (1) predict general remission across antidepressants and (2) differentially predict remission by type of antidepressant. We also assessed whether pretreatment functional connectivity, as a function of remission status, is abnormal relative to controls in remitters or non-remitters and whether connectivity changes from pretreatment to posttreatment. Given that both hyper- and hypo-connectivity within and between the DMN has been documented in the literature (e.g., refs ^11,22,23^) and the dearth of studies examining intrinsic functional connectivity predictors of depression response, we did not have specific directional predictions related to aims 1 nor 2.

## Materials/subjects and methods

### Overview and study design

Functional connectivity data were obtained from 80 participants with MDD and from 34 healthy controls from the iSPOT-D (See Supplemental Figure S1 for the full CONSORT chart). A complete description of the randomized iSPOT-D practical trial protocol, clinical assessments, inclusion/exclusion criteria, and diagnostic procedures is provided in ref. ^21^. In short, the primary diagnosis of non-psychotic MDD was confirmed using the Mini-International Neuropsychiatric Interview (MINI)^24^, according to Diagnostic and Statistical Manual of Mental Disorders, Fourth Edition criteria, and inclusion criteria included a score ≥16 on the 17-item Hamilton Rating Scale for Depression (HRSD_17_)^25^. Exclusion criteria included multiple comorbidities including current or past diagnosis of psychosis, bipolar disorder, posttraumatic stress disorder, obsessive compulsive disorder, and any Axis II personality disorder as assessed by the MINI or any medical condition that might interfere with administration of assessments or the safety of antidepressant medication. Sample size was chosen as part of the original protocol development in order to achieve statistical power of 80% at an effect size of 1 standard deviation^21^.

This study was conducted according to the principles of the Declaration of Helsinki 2008. Participants provided written informed consent after the study procedures were fully explained in accordance with the ethical guidelines of the institutional review board.

All participants were either antidepressant medication naive or, if previously prescribed an antidepressant medication, had undergone a wash-out period of at least 1 week (five half-lives). Participants were randomized to receive sertraline, escitalopram, or venlafaxine-XR using PhaseForward’s validated, Web-based Interactive Response Technology as previously described^26^. For further details, see supplemental methods.

### Criteria for treatment outcomes

The primary study outcome was treatment remission defined as a score of ≤7 on the HRSD_17_ using clinician ratings from the HRSD_17_ scale as this was the primary outcome measure of the iSPOT-D protocol^21^. We analyzed data for participants who completed the posttreatment scanning session, consistent with previous studies that included a posttreatment scan^27,28^.

### Study treatments

A blocked randomization procedure was undertaken centrally for the full iSPOT-D study (block size of 12, across sites). The imaging was conducted solely at the Sydney site. Investigators, raters, and participants were not blind to treatment assignment. The medication doses were prescribed and adjusted by treating clinicians according to routine clinical practice and followed the recommended dose ranges.

Any treatment for concurrent general medical conditions were allowed and recorded. Comorbid general medical conditions were recorded under the categories (with examples) of cardiovascular (hypertension), digestive (irritable bowel syndrome), endocrine (diabetes), hemic/lymphatic (gout), metabolic/nutritional (high cholesterol), musculoskeletal (tendonitis), respiratory (asthma), urogenital (kidney stone), skin (eczema), and special senses (astigmatism) disorders. Approximately 50% of the sample reported no comorbid general medical condition in these categories, 23% reported one condition, and 27% one or more conditions.

Participants discontinued psychotropic medication prior to randomization with the exception of occasional (i.e., ⩽1 dose/week) use of anxiolytics, sleep aids, and medications to manage anti- depressant-induced side effects (e.g., nausea) as they reflect common practice. In all, 4.9% of patients within the total sample were taking a concomitant psychotropic medication, including the anxiolytic Alprazolam and the sedative/hypnotics, Eszopiclone, Triazolam, Zolpidem, and Zopiclone.

### Image acquisition

Magnetic resonance imaging (MRI) images were acquired in Sydney, Australia using a 3.0-T GE Signa scanner and an eight-channel head coil. The intrinsic functional connectivity data were acquired using a previously validated approach^29^. Specifically, the scan consisted of five tasks and a three-dimensional (3D) T1-weighted structural MRI scan. MR images for each task were acquired using echo planar imaging (TR = 2500 ms, TE = 27.5 ms, matrix = 64 × 64, FOV = 24 cm, flip angle = 90 degrees). Forty slices, each 3.5 mm thick, covered the whole brain in each volume. For each task, 120 volumes were collected with a total scan time of 5 min and 8 s. The details of the five tasks have been previously described^30^. Briefly, tasks assessed (1) selective attention using an auditory oddball task, (2) working memory using a continuous performance task, (3) inhibition processes using a Go-NoGo task, and (4) conscious and (5) non-conscious processing of emotional faces. Intrinsic functional connectivity was derived from the residual time series when all five tasks were concatenated, following the removal of task and covariate effects (more details below). While this method is distinct from methods used to assess resting-state connectivity in which there is no specific task context and participants are engaged in non-directed attention, this procedure results in patterns of functional connectivity that closely mimic those found in resting-state scans^30^ and can also be considered to assess the “task negative” nature of the DMN^8,31^. We believe that this approach may hold more ecological validity than standard resting-state paradigms, given that individuals switch from free thought/self-reflection to goal-directed task states in daily life, and do not necessarily have extended periods of resting non-directed thought.

Structural MRI 3D T1-weighted images were acquired in the sagittal plane using a 3D spoiled gradient echo sequence (TR = 8.3 ms; TE = 3.2 ms; flip angle = 11 degrees, TI = 500 ms, NEX = 1, ASSSET = 1.5, matrix = 256 × 256). A total of 180 contiguous slices, each 1 mm thick, covered the whole brain with an in-plane resolution of 1 × 1 mm^2^.

### Preprocessing of connectivity data

Processing of intrinsic functional connectivity was assessed using an established imaging procedure^30^. Statistical Parametric Mapping software implemented in MATLAB (SPM8; Wellcome Department of Cognitive Neurology, London) was used for the preprocessing and data analysis. First images were motion corrected and unwarped using default parameters in SPM8. Following realignment and unwarping, quality-control diagnostics were completed on the time series data for each run. Data volumes that were associated with extreme (1) movement (framewise displacement from one time point to the next) and (2) changes in blood-oxygen-level-depedent BOLD signal intensity (as indexed by the mean squared difference in signal intensity over the entire volume from one time point to the next divided by the mean signal across the volume averaged across the full time series) were censored (temporally masked) to reduce the influence of motion and related artifacts. Framewise displacement was calculated as the sum of the absolute values of the differentiated realignment estimates as in Power et al. (2014)^32^. Volumes were censored using established thresholds of framewise displacement ⩾0.3 mm and scaled signal intensity differences >10^32–35^. Censoring was implemented with the time series difference analysis toolbox http://www.fil.ion.ucl.ac.uk/spm/ext/# TSDiffAna and in-house scripts. A temporal mask was then created for each censored volume (as well as subsequent volume) and used as regressors of no interest in the first-level statistical models^32,34^. Since movement-related artifacts have been shown to impact volumes acquired before and several seconds after a movement spike, to reduce the influence of movement-related artifacts a total of four temporal masks were created for each movement spike (an additional volume before and two volumes after the movement spike)^32^. Further, participants who had >200 volumes temporally masked (1/3 of the total intrinsic connectivity time course) were removed from group analyses. Images were then slice time corrected in SPM8. Following slice time correction, images were spatially normalized to the stereotactic Montreal Neurological Institute (MNI) space using the FMRIB nonlinear registration tool^36^ and smoothed using an 8 mm full-width-at-half-maximum Gaussian kernel in SPM8.

For each functional MRI (fMRI) task, general linear models (GLMs) were used to model the BOLD responses for each experimental condition: oddball (target and nontarget trials), continuous performance (working memory, 1-back and baseline trials), Go-NoGo tasks (Go and NoGo trials), and both emotion tasks (each emotion type). Motion effects were also modeled for each task using the Volterra expansion of the realignment parameters proposed in Friston et al. (1996) (24 regressors; *R*_*t*_, *R*_*t*_^2^, *R*_*t*-1_, *R*_*t*-1_^2^ where *R*s are the realignment parameters estimated during the preprocessing stage)^37^. Additional covariates for each task included the mean signal time course extracted from eroded ventricle and white matter masks as well as the temporal masks derived from the volume censoring described above. The intrinsic functional connectivity signal was estimated as the residual images after modeling the BOLD signal for each stimulus of the above tasks as repressors of non-interest. After this, a band-pass filter (0.009 < *f* < 0.08 Hz) was applied.

### Connectivity analysis

Connectivity within the DMN was identified using a seed-based correlation approach. The average BOLD time series was extracted from two bilateral 5-mm spheres centered on the PCC coordinates from Gordon et al. ([*x*, *y*, *z*]: [Left: −11, −52, 37; Right: 12, −52, 35])^38^. The resultant time series was then used as a regressor of interest in a first-level model in SPM8 to generate a correlation map for each subject. The resulting correlations were then transformed into *z*-scores using the Fisher-*z* transform and entered into second-level models.

### Primary analysis of general prediction

Step 1: To identify regional clusters where pretreatment intrinsic functional connectivity was associated with posttreatment antidepressant outcomes independent of antidepressant type, we implemented a GLM in SPM8 with treatment outcome (remitter versus non-remitter at 8 weeks posttreatment) as the between-subjects factor. Given that our primary aim was to identify intrinsic functional connectivity clusters that differed by remissions status, as opposed to MDD diagnosis status, we chose to exclude healthy controls from this portion of the analysis. Because non-remitter and remitter subgroups differed slightly on the severity of depressive symptoms (HRSD_17_), MDD episode duration, and age, these variables were included as covariates. Clusters were retained for additional analyses if they met a conservative family-wise error (FWE)-cluster corrected *p* < 0.001 (one-sided) to correct for multiple comparisons. An additional supplementary whole-brain, voxel-wise analysis was conducted using the continuous variable of the percent reduction of depression severity with the same covariates and FWE-cluster corrected threshold as above.

Step 2: To undertake our planned contrasts, *β* weights were extracted from the voxels that showed significant connectivity with the PCC seed in Step 1. These *β* weight data were analyzed using R. We compared differences in PCC intrinsic functional connectivity between (1) remitters and healthy controls and (2) non-remitters and healthy controls using multiple regression analyses with PCC connectivity as the dependent variable, group membership as a dummy coded variable, and age entered as a covariate. We chose to use these planned *t*-tests to compare each group separately to controls rather than an analysis of variance framework since it is possible that one of the remission subgroups may resemble that of controls. Following the procedure of Williams et al. (2015)^26^, separate paired *t*-tests were used to assess changes from baseline to follow-up visit in each group within the same intrinsic functional clusters.

### Analysis of differential prediction

Step 1: To determine whether individuals who remitted to specific antidepressant treatments were also differentiated by pretreatment intrinsic functional, we again used a GLM (in SPM8) with a second between-subjects factor for each antidepressant type (escitalopram, sertraline, and venlafaxine-XR). Covariates again included clinician-rated depression severity at baseline, MDD episode duration, and age. Here we tested the interaction between remission status and antidepressant medication. Following our procedure for testing general prediction, the primary focus of our analysis was on voxel-wise FWE-cluster corrected *p* < 0.001 (one-sided) level threshold. Clusters were retained for secondary analyses if they met this FWE-cluster corrected *p* < 0.001 to correct for multiple comparisons. An additional supplementary whole-brain, voxel-wise analysis was conducted using the continuous variable of the percent reduction of depression severity with the same covariates and FWE-cluster corrected threshold as above.

Step 2: *β* weights were extracted from the voxels that defined the cluster of significant connectivity with the PCC seed identified in Step 1, and GLMs were then used to compare the connectivity of healthy controls to that of remitters and non-remitters within each medication type, with age as a covariate. Following the procedure of Williams et al. (2015)^26^, separate paired *t*-tests were used to assess changes from baseline to follow-up visit in each group within the same intrinsic functional cluster.

### Classification sensitivity and specificity of PCC–ACC/mPFC connectivity

Given our a priori hypotheses that connectivity between the PCC and ACC/mPFC would be predictive of treatment outcome, we conducted a secondary analysis using hierarchical logistic regression and receiver operating characteristic (ROC) analyses implemented in R to characterize the contribution of the PCC– ACC/mPFC connectivity in the classification of remission status. Specifically, connectivity was extracted from a mask centered on ACC/mPFC coordinates identified using a meta-analytic approach (see below), mean centered, scaled by the standard deviation, and entered as a predictor of remission status. In addition to identifying an ACC/mPFC cluster that may survive correction for multiple comparisons described above, we considered it also important to demonstrate the strength of the prediction using an independently generated region of interest (ROI). This approach avoids issues of double dipping^39^, which can lead to increased bias and reduced generalizability. ROC curves were created with the Epi package in R^40^. The Wald Statistic (W) was used to determine the significance of the contribution of each predictor.

### Cross-validation analyses

To increase the generalizability of the model predictions and reduce the bias caused by model fitting^41,42^, predictive performance of the ROC analyses was also examined using leave-one-out cross-validation.

### Specificity of prediction of remission by PCC-ACC/mPFC connectivity

To determine whether intrinsic functional connectivity may be serving as a proxy for other behavioral and patient characteristics previously associated with the prediction of remission outcomes in depressed patients, we conducted supplementary linear regression analyses between intrinsic functional connectivity within the significant clusters defined by Step 1 and baseline anxiety scores (anxiety subscale of the HRSD^3,25,43^), presence of comorbid anxiety diagnosis^3^, number of early life events^42,44^, body mass index^45^, and cognitive impairment^46^. Additional supplementary logistic regression analyses tested whether PCC–ACC/mPFC connectivity would add additional predictive power over behavioral and patient characteristics that have previously been associated with remission status.

### Defining ACC/mPFC ROI mask

The ACC/mPFC ROI as defined based on an automated meta-analysis of 516 studies using NeuroSynth (www.neurosynth.org)^47^ was conducted on (May, 26th 2017) with the search term “Default Mode.” NeuroSynth uses automated, text-based data mining on abstracts of published neuroimaging studies to derive meta-analytic, whole-brain maps for >3000 search terms^47^. The coordinate with the peak *Z* score within the mPFC (*x* = −2, *y* = 50, *z* = −6) was identified using AFNI’s 3dExtrema function on the forward inference map. To ensure that only voxels that were part of the original NeuroSynth map were included, and to maintain regional specificity within the ACC/mPFC, a mask was derived by taking the overlap of the original forward inference NeuroSynth map and a 10 mm radius spherical mask centered on the identified peak coordinates of the ACC/mPFC.

**Fig. 1:**
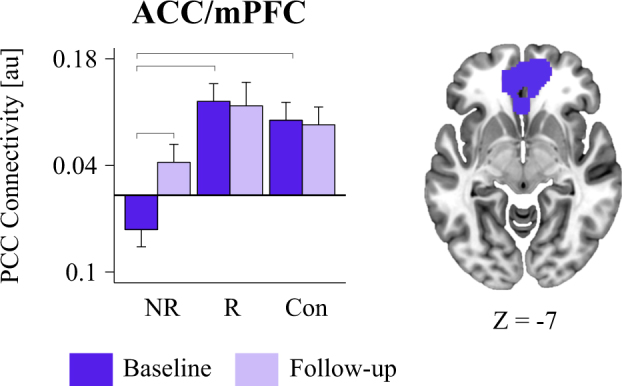
The brain maps display the binary mask for the ACC/mPFC cluster identified in the voxel-wise comparison of remitters versus non-remitters. The bar graph illustrates the difference across groups (remitters, non-remitters, and healthy controls) and sessions (baseline and 8-week follow-up) in functional connectivity with the PCC seed with the ACC/mPFC cluster. The ACC/mPFC cluster showed greater connectivity in the remitters compared to the non-remitters. Brackets across bars denote significance *p* < 0.05 in the planned comparisons. ACC anterior cingulate cortex, mPFC medial prefrontal cortex, PCC Posterior Cingulate Cortex, NR Non-Remitters, R Remitters, Con Controls

### Code availability

The code used to generate the results for the current study is available from the corresponding author on reasonable request.

## Results

### Participant characteristics

Following the established iSPOT-D imaging protocol, 80 MDD patients completed fMRI scans before and 8 weeks after randomization to escitalopram, sertraline, or venlafaxine-XR^48^. Thirty-four healthy controls similarly were scanned at equivalent time points. Four patients (three who received escitalopram and two who received venlafaxine) and two controls were subsequently removed due to excessive movement within the scanner, and one patient and one control were excluded due to incomplete scans. Table 1A, B provide clinical and demographic characteristics of each group. Because remitters and non-remitters differed on duration of MDD (*t* = −2.60, *p* = 0.011) and age (*t* = −2.63, *p* = 0.011), these variables along with pretreatment depression severity were included as covariates.

**Table 1A:** Demographic and clinical characteristics by group

Characteristic	Non-remitters			Remitters			All MDD			Healthy controls		
	Mean	SD	*n*	Mean	SD	*n*	Mean	SD	*n*	Mean	SD	*n*
Age of first visit	34.98	13.69	38	28.34	7.10	37	31.70	11.38	75	29.93	10.91	31
Years of education	14.00	3.14	38	14.65	2.41	37	14.32	2.80	75	14.84	2.72	31
Duration of illness	14.61	13.26	38	8.32	6.48	37	11.50	10.88	75	—	—	—
Number of prior episodes	19.95	11.93	38	19.54	7.70	37	19.75	10.00	75	—	—	—
Body mass index	25.31	4.91	32	26.16	6.29	35	25.75	5.65	67	—	—	—
Attention	−0.04	0.86	33	0.03	0.43	35	0.00	0.67	68	0.09	0.46	30
Cognitive flexibility	0.36	1.27	33	−0.01	0.73	35	0.17	1.04	68	0.05	0.50	30
Decision speed	−0.17	1.69	33	−0.02	0.57	35	−0.09	1.24	68	−0.08	0.77	30
Executive functioning	0.20	0.97	33	−0.19	1.4	35	0.00	1.22	68	0.15	0.47	26
Information processing speed	0.17	0.77	33	0.08	0.54	34	0.12	0.66	67	0.11	0.53	30
Motor coordination	−0.12	0.36	33	−0.12	0.45	35	−0.12	0.41	68	−0.06	0.58	30
Response inhibition	−0.07	0.72	32	−0.01	0.43	35	−0.04	0.58	67	0.07	0.53	29
Verbal memory	−0.15	0.73	33	0.09	0.9	35	−0.03	0.83	68	0.02	0.82	30
Working memory	−0.24	1.09	33	−0.03	1.07	35	−0.14	1.08	68	0.23	1.12	30
Number of early-life stressors	4.34	2.09	35	3.22	2.69	37	3.76	2.46	72	0.88	1.28	26
HRSD_17_ total Sscore at baseline	20.5	3.32	38	21.78	4.34	37	21.13	3.88	75	1.10	1.30	30
HRSD_17_ anxiety score at baseline	6.87	2.06	38	6.97	1.89	37	6.92	1.96	75	0.45	0.63	29
QIDS-SR_16_ total score at baseline	13.67	3.39	36	14.08	4.02	37	13.88	3.70	73	2.23	1.77	26
Dosage												
Escitalopram	15.00	11.68	12	10.00	4.26	12	12.50	8.97	24	—	—	—
Sertraline	62.50	25.48	14	55.77	29.14	13	59.26	26.99	27	—	—	—
Venlafaxine	100.00	36.93	12	81.25	21.65	12	90.62	31.11	24	—	—	—
Equivalent dosage^a^												
Escitalopram	112.50	87.58	12	75.00	31.98	12	93.75	67.26	24	—	—	—
Sertraline	93.75	38.21	14	83.65	43.72	13	88.89	40.48	27	—	—	—
Venlafaxine	100.00	36.93	12	81.25	21.65	12	90.62	31.11	24	—	—	—

^a^Equivalent dosage in venlafaxine (7.5 × escitalopram; 1.5×sertraline; 1×venlafaxine)

**Table 1B:** Demographic and clinical characteristics by group

Characteristic	Non-remitters	Remitters	All MDD	Controls
	*n*	*n*	*n*	*n*
Sex				
Male	19	19	38	16
Female	20	17	37	15
Comorbid anxiety diagnosis				
Yes	16	19	35	—
No	22	18	40	—

### General prediction of remission by connectivity with the PCC

#### Step 1: General prediction by connectivity between the PCC node of the DMN and the ACC/mPFC

At the pretreatment baseline, PCC connectivity with a cluster spanning regions of the ACC/mPFC, distinguished patients who subsequently went on to remit after 8 weeks of antidepressants from those who did not remit at the FWE threshold. The ACC/mPFC is a defining region of the anterior DMN. Remitters were distinguished from non-remitters by relatively intact connectivity between the PCC and ACC/mPFC and were not distinguishable from healthy controls. (Table 2; Fig. 1 and Supplementary Figure S2). A supplemental whole-brain, voxel-wise analysis revealed similar associations between the continuous variable of symptom reduction and PCC–ACC/mPFC (Supplementary Figure S2).

**Table 2:** General treatment prediction (HAM-D remission)

Region (BA)	Step 1: Significant prediction effects							Step 2: Follow-up comparisons	
	FWE-Corr (Cluster)	Cluster size (# of voxels)	*x*	*y*	*z*	*Z* score (Peak)	Cohen’s *d*^a^ (Cluster)	Pretreatment comparison to controls (Cluster)	Change from pretreatment to posttreatment (Cluster)
Remitters>non-remitters									
ACC/mPFC (BA 24)-L	**0.000**	2880	−4	32	6	4.52	**R>NR** ***d*** **=** **1.16**	**NR<C:** ***p*** **=** **0.000,** ***d*** **=** **1.22** R = C: *p* = 0.943, *d* = 0.02	**NR: Pre>Post,** ***p*** **=** **0.006** R: Pre = Post, *p* = 0.832 C: Pre = Post, *p* = 0.486

Columns under step 1 are referring to voxel-wise results from a whole-brain fMRI analysis using a family-wise error corrected *p*-value for clusters at alpha *p* < 0.001 cluster extent threshold. Columns under Step 2 are referring to the statistics calculated on the extracted beta estimates from the clusters identified in Step 1

Bold values denote significant effects

*FWE-Corr* SPM8 family-wise error corrected *p*-value for clusters at alpha *p* < 0.001 correcting for multiple comparisons, *cluster size* the number of voxels within the cluster that survived threshold, *x*, *y*, *z* coordinates in MNI space of the peak voxel identified in the cluster, *Z* *score* the *Z* score of the peak voxel within the cluster, *Cohen’s* *d* for the extracted beta coefficients of the full cluster identified in the voxel wise analysis, *BA* Brodmann’s area; *L* left, *ACC* anterior cingulate cortex, *mPFC* medial prefrontal cortex, *R* remitters, *NR* non-remitters, *C* controls

^a^Controlling for age, duration of depressive episode, and baseline depression severity

**Fig. 2:**
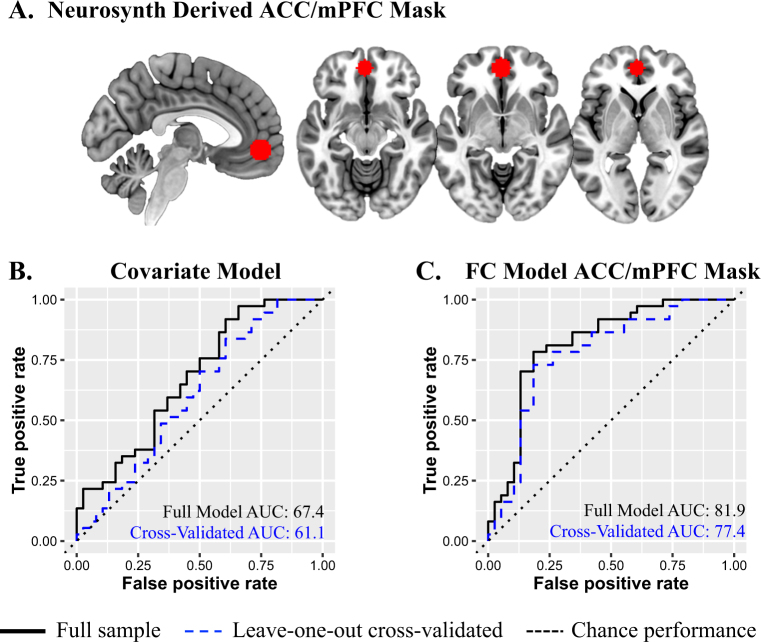
**a** NeuroSynth ACC/mPFC mask used to extract connectivity with PCC for ROC analyses. ROC curve model performance in predicting remission for 75 participants using the following predictors: **b** demographic/clinical measures (including age, MDD duration, and baseline depression severity) and **c** demographic/covariate measures and the connectivity between the PCC seed and the NeuroSynth-defined ACC/mPFC mask. AUC area under the curve, ROC receiver operating characteristic, FC functional connectivity, ACC anterior cingulate cortex, mPFC medial prefrontal cortex

#### Step 2: Characterizing if connectivity predictors distinguish patients from controls at baseline and change from baseline to posttreatment

Planned comparisons revealed that remitters did not differ from controls in pretreatment intrinsic functional connectivity in the ACC/mPFC cluster (*p* > 0.94, *d* < 0.02). However, non-remitters (unlike remitters) differed substantially from controls at baseline, showing comparative *hypo*-connectivity of the PCC with anterior DMN cluster (ACC/mPFC: *p* < 0.001, Cohen’s *d* = 1.22; Table 2; Fig. 1). Despite lack of remission, hypo-connectivity tended to increase at follow-up (ΔACC/mPFC: *p* < 0.006; Table 2; Fig. 1. There were no changes from pretreatment to posttreatment for controls in the ACC/mPFC cluster (*p* = 0.486).

### Differential prediction of remission by connectivity with the PCC

#### Step 1. Differential prediction by connectivity with the PCC node of the DMN

No clusters were identified in the differential prediction of remission according to antidepressant type at the FWE threshold. Similarly, no voxels survived correction in the whole-brain, voxel-wise analysis using the continuous variable of symptom reduction.

### Specificity of prediction of remission by PCC–ACC/mPFC connectivity

Previous studies, including those with the participants from the iSPOT-D dataset, have identified other patient characteristics and biomarkers that predict remission outcomes, including comorbid anxiety^3^, exposure to early life trauma^42,44^, body mass index^42^, and cognitive impairment^46^. Thus, in supplementary linear regression analyses, we considered whether the role of intrinsic connectivity in predicting remission status may reflect differences in these predictor variables at baseline or instead contribute independently to prediction beyond these other factors. At pretreatment baseline, we found that ACC/mPFC connectivity cluster was not associated with any of these variables (Supplemental Table S1). PCC–ACC/mPFC intrinsic functional connectivity also predicted remission above and beyond any of these previously identified biomarkers (Supplemental Table S2). Thus intrinsic functional connectivity is likely to be an important independent contributor to baseline characteristics that predict remission outcomes.

### Classification accuracy of PCC–ACC/mPFC connectivity

The ROI mask identified using our neurosynth procedure contained 498 voxels (Fig. 2a). The connectivity between the PCC and ACC/mPFC together with the covariates of age, duration of MDD, and pretreatment depression severity significantly predicted remission status (Δ*χ*^2^ = 26.79, Δdf = 4, *p* < 0.0001). Importantly, the PCC–ACC/mPFC connectivity contribution to classification was significant beyond that of a model solely consisting of covariates (Δ*χ*^2^ = 16.55, Δdf = 1, *W* = 3.505, *p* < 0.001). ROC analyses revealed high predictive accuracy, sensitivity, and specificity for both the full model (81.9%, 78.4%, and 82.0%, respectively; Fig. 2c) and the leave-one-out cross-validated model (77.4%, 73.0%, and 81.6%; Fig. 2c), suggesting that this model may be generalizable. Including the intrinsic functional predictor increased classification model accuracy by 16.9% as compared to a model with the covariates alone (cross-validated accuracy = 60.5, sensitivity = 83.8%, specificity = 39.5%; Fig. 2b).

## Discussion

In this study, we demonstrate that knowing about intrinsic functional connectivity of the brain prior to commencing treatment may be a clinically applicable predictor, with 75% accuracy, for assessing which patients with depression are likely to benefit from a first-line antidepressant and which patients are not. Specifically, we found that functional connectivity between the posterior and anterior regions of the DMN, a network previously associated with depression pathophysiology, predicts clinical remission following antidepressant treatment. When connectivity was disrupted, it predicted poorer treatment outcomes. This predictive relationship was observed for outcomes defined by remission and the magnitude of symptom change. We have confidence that these results are generalizable across definitions of the DMN, as predictive accuracy remained high (75%) when examining connectivity based on regions defined by a meta-analytic approach.

A striking finding in the present study was that, at pretreatment, the profile of posterior–anterior DMN connectivity in eventual remitters closely resembled that of healthy controls. One potential interpretation of this resemblance is that the intrinsic functional organization of DMN connections must be substantially intact in order to confer the neural capacity to mount a clinically significant acute response to antidepressant treatment. A further implication is that utilizing alternative interventions that have the capacity to directly alter intrinsic functional connectivity within these networks such as transcranial magnetic stimulation prior to beginning antidepressant treatment may aide in remediating symptoms in those individuals with dysfunctional connectivity. Critically, the absence of associations between posterior–anterior DMN connectivity and several other variables that had been previously associated with depression course suggests that the intrinsic functional connectivity may provide additional information about treatment outcome that would not be available through other clinical, behavioral, or demographic measures. This profile of abnormal pretreatment connectivity in non-remitters attenuated after 8 weeks of follow-up despite the lack of clinical remission. Because attenuation of abnormality was in the direction of taking the patients closer to healthy controls, it is possible that non-remitters would in fact remit on a longer course of treatment. It is also possible that factors independent of treatment or clinical characteristics drive the profiles of functional connectivity that distinguish non-remitters from both remitters and controls. These possibilities require further investigation, including in a study of longer-term treatment phases.

Our finding that intrinsic connectivity in the pretreatment state may differ as a function of subsequent treatment outcome might help disentangle the question of how and to what extent disrupted connectivity is a common characteristic of depressive disorder itself. Some studies have reported that MDD is characterized by hypo-connectivity of posterior and anterior regions of the DMN relative to controls (e.g., refs ^22,23^), while others suggest hyper-connectivity (e.g., ref. ^11^) and yet others, no difference^10,20^. The results from the current study together with prior findings indicating unique subtypes of depression that are defined by the presence or absence of PCC–mPFC connectivity^20^ suggest that MDD may in fact comprise multiple intrinsic connectivity types, including some patients who have disrupted connectivity as well as others who do not, and that these types may be important for treatment outcome.

Previously studies have typically estimated intrinsic functional connectivity using a resting-state scan that is distinct from task conditions. Here we estimated intrinsic connectivity extracted from regular periods of rest within a standardized series of cognitive and emotional task conditions. This procedure was developed based on principles of ecological validity to mimic participants switching between rest and task in their natural world functioning. This procedure has been shown to yield intrinsic default mode connectivity that is highly correlated with “stand-alone” resting condition connectivity^30^ and is internally consistent between each contributing task condition^30,31^. However, given evidence that functional connectivity elicited by task conditions can persist into periods of rest^49^, future studies should compare our results to those using more traditional stand-alone resting-state scans.

These findings should be appreciated within the context of certain limitations. Owing to the practical trial design, antidepressants in the study were limited to those in common use at each treatment site. It would be important for future studies to verify whether functional connectivity also predicts remission when using other antidepressants and second-generation antipsychotics with antidepressant properties that have distinct mechanisms of action, as well as psychotherapy. Similarly, it would be important to evaluate whether different disruptions to intrinsic functional connectivity differentially predict outcomes for multiple different active treatments. Moreover, in this study treatment outcome was only assessed at the 8-week time point. Including multiple follow-ups before and after the 8-week time point would be important to determine whether predictive relationships change as a function of time during treatment.

In conclusion, our results advance the understanding of the contribution of functional connectivity to the pathophysiology of MDD and response to antidepressant treatment. We show, in a relatively large treatment sample, that pretreatment functional connectivity profiles hold promise for developing neuroscience-informed approach to mental disorder and its management.

## Electronic supplementary material

Supplemental Figure S1

Supplemental Figure S2

Supplemental Table S1

Supplemental Table S2

Supplemental Figure Legends
