# Supplementary material for: Intrinsic functional connectivity predicts remission on antidepressants: a randomized controlled trial to identify clinically applicable imaging biomarkers
Source: Transl Psychiatry. 2018 Mar 6;8:57. doi: 10.1038/s41398-018-0100-3 (PMC5838245; doi:10.1038/s41398-018-0100-3)
Supplement: Supplementary file 1 — Supplemental Figure S1 [file 41398_2018_100_MOESM1_ESM.pdf]

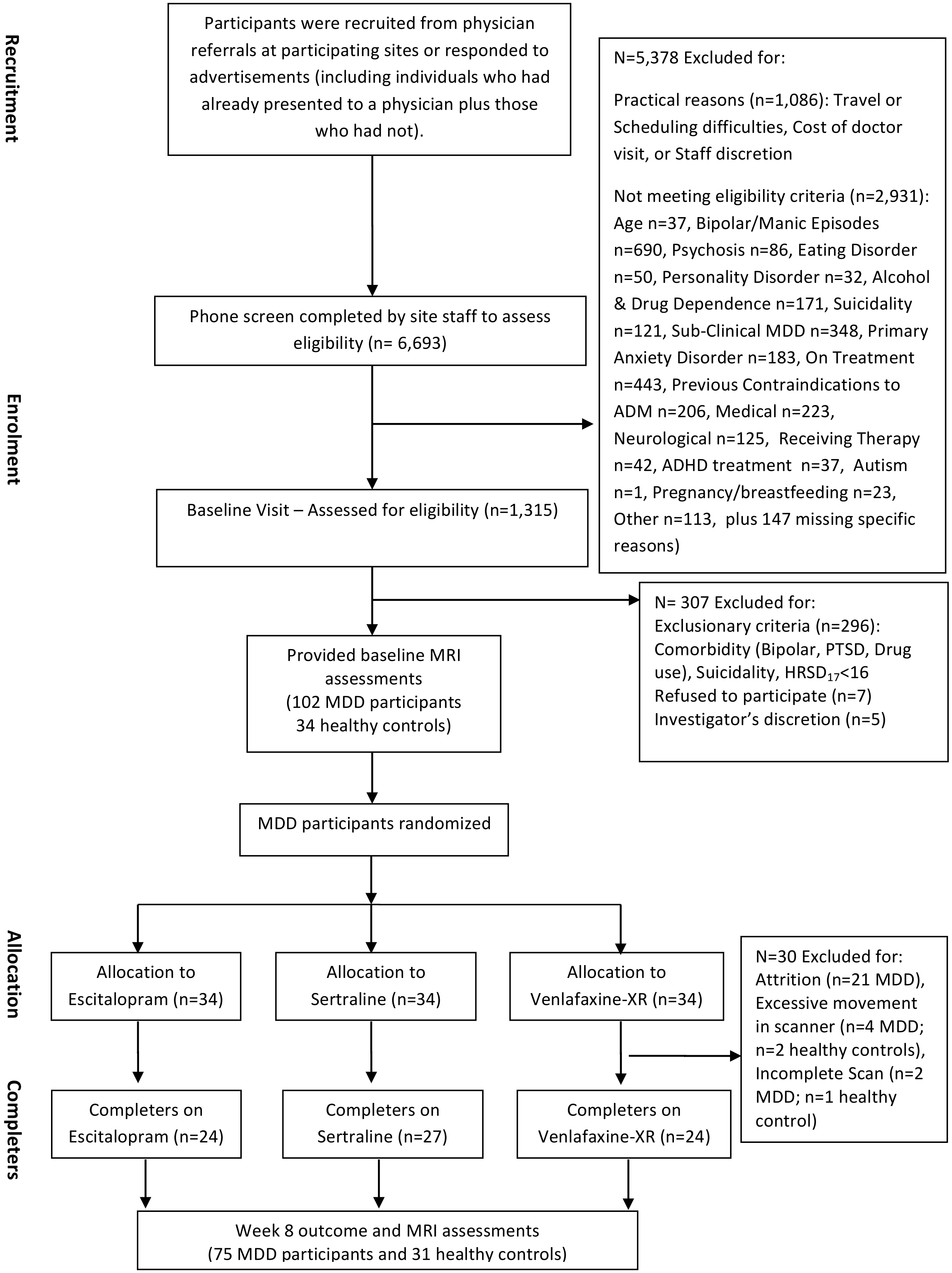

Abbreviations: ADHD: Attention Deficit Hyperactivity Disorder, ADM: antidepressant medication, $HRSD_{17}$, 17-item Hamilton Rating Scale for Depression; MDD: Major Depressive Disorder; MRI: Magnetic Resonance Imaging; PTSD, Post-Traumatic Stress Disorder
