# Supplementary material for: Intrinsic functional connectivity predicts remission on antidepressants: a randomized controlled trial to identify clinically applicable imaging biomarkers
Source: Transl Psychiatry. 2018 Mar 6;8:57. doi: 10.1038/s41398-018-0100-3 (PMC5838245; doi:10.1038/s41398-018-0100-3)

**A.** Regions in which Remitters > Non-Remitters in connectivity

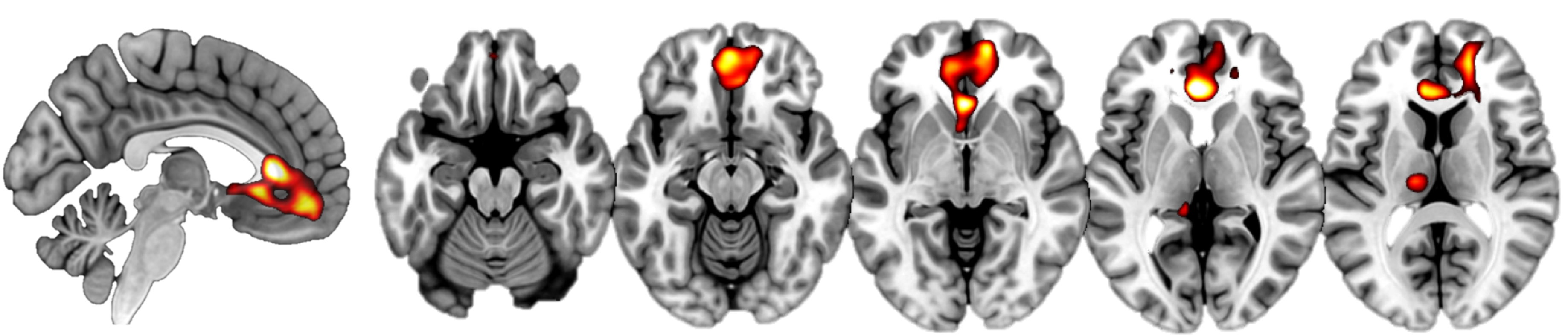

**B.** Regions that showed a linear association with $HRSD_{17}$ Percent Reduction from Baseline

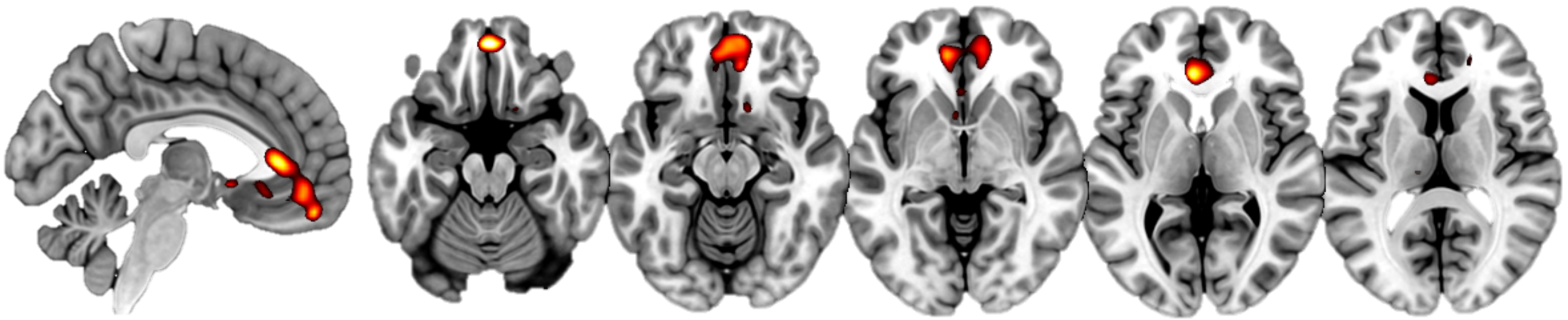

**C.** Overlap between binary and linear associations with treatment outcome

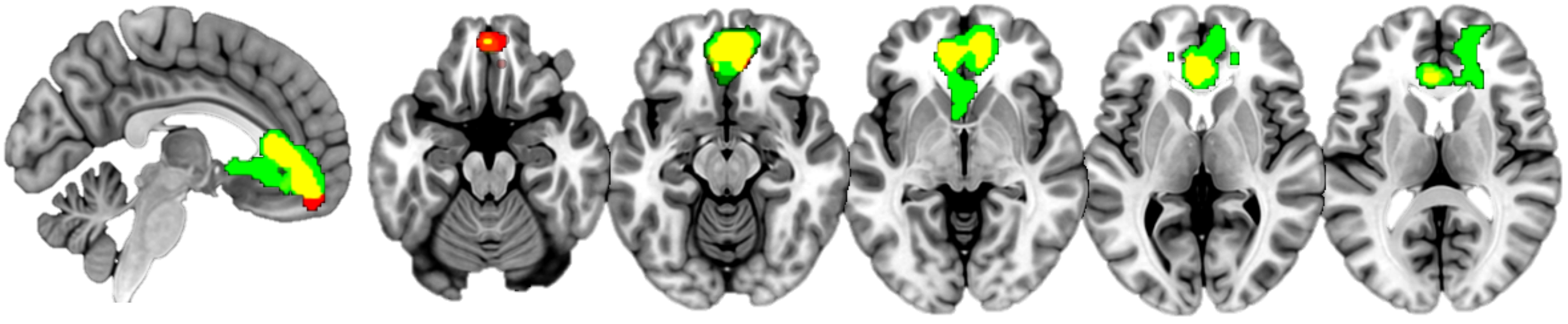

Supplement: Supplementary file 2 — Supplemental Figure S2 [file 41398_2018_100_MOESM2_ESM.pdf]
