# Supplementary material for: Intrinsic functional connectivity predicts remission on antidepressants: a randomized controlled trial to identify clinically applicable imaging biomarkers
Source: Transl Psychiatry. 2018 Mar 6;8:57. doi: 10.1038/s41398-018-0100-3 (PMC5838245; doi:10.1038/s41398-018-0100-3)
Supplement: Supplementary file 3 — Supplemental Table S1 [file 41398_2018_100_MOESM3_ESM.docx]

| **Table S1. Associations between Demographic/Clinical Characteristics and PCC-ACC/mPFC Connectivity** | | | | | |
| --- | --- | --- | --- | --- | --- |
| **Characteristic** | **B (95% CI)** | **SE** | **t-value** | **p-value** | **R^2^** |
| HDRS_17_ Anxiety | -0.01 (-0.03-0.02) | 0.01 | -0.72 | 0.473 | 0.01 |
| Comorbid Anxiety Diagnosis | -0.07 (-0.16-0.02) | 0.05 | -1.49 | 0.140 | 0.03 |
| Number of Early Life Stressors | 0.00 (-0.02-0.02) | 0.01 | -0.18 | 0.859 | 0.00 |
| Body Mass Index | 0.01 (0.00-0.01) | 0.00 | 1.28 | 0.206 | 0.02 |
| Cognition |  |  |  |  |  |
| Attention | 0.00 (-0.07-0.07) | 0.04 | -0.02 | 0.987 | 0.00 |
| Cognitive Flexibility | -0.02 (-0.07-0.02) | 0.02 | -0.94 | 0.352 | 0.01 |
| Decision Speed | 0.01 (-0.03-0.05) | 0.02 | 0.71 | 0.480 | 0.01 |
| Executive Functioning | -0.03 (-0.07-0.01) | 0.02 | -1.38 | 0.173 | 0.03 |
| Information Processing Speed | 0.03 (-0.04-0.10) | 0.04 | 0.80 | 0.429 | 0.01 |
| Motor Coordination | 0.01 (-0.11-0.12) | 0.06 | 0.09 | 0.929 | 0.00 |
| Response Inhibition | -0.01 (-0.09-0.08) | 0.04 | -0.18 | 0.861 | 0.00 |
| Verbal Memory | 0.01 (-0.05-0.07) | 0.03 | 0.40 | 0.689 | 0.00 |
| Working Memory | 0.04 (0.00-0.08) | 0.02 | 1.80 | 0.077 | 0.05 |
|  |  |  |  |  |  |
|  |  |  |  |  |  |
|  |  |  |  |  |  |
