# Supplementary material for: Intrinsic functional connectivity predicts remission on antidepressants: a randomized controlled trial to identify clinically applicable imaging biomarkers
Source: Transl Psychiatry. 2018 Mar 6;8:57. doi: 10.1038/s41398-018-0100-3 (PMC5838245; doi:10.1038/s41398-018-0100-3)
Supplement: Supplementary file 4 — Supplemental Table S2 [file 41398_2018_100_MOESM4_ESM.docx]

**Table S2. ACC-mPFC Connectivity Predicting HRSD_17_ Remission with additional Demographic and Clinical variables**

| **Model Predictors** | **B (95% CI)** | **SE** | **Wald** | **p-value** | | **Model Fit** | | | |  |
| --- | --- | --- | --- | --- | --- | --- | --- | --- | --- | --- |
|  |  |  |  |  | | **χ^2^(df)** | | ***p*-value** | |  |
| HRSD_17_ Anxiety |  |  |  |  |  | |  | | | |
| Intercept | -1.33 (-4.95-2.13) | 1.78 | -0.75 | 0.456 | | 95.94(5) | | | 0<0.0001 |  |
| Age | -0.02 (-0.09-0.04) | 0.03 | -0.72 | 0.471 | |  | | |  |  |
| MDD Duration | -0.08 (-0.18-0.00) | 0.05 | -1.70 | 0.089 | |  | | |  |  |
| HRSD_17_ | 0.16 (-0.02-0.37) | 0.10 | 1.68 | 0.093 | |  | | |  |  |
| HRSD_17_ Anxiety | -0.22 (-0.63-0.16) | 0.20 | -1.09 | 0.278 | |  | | |  |  |
| **PCC-ACC/mPFC Connectivity** | **5.96 (2.84-9.69)** | **1.73** | **3.45** | **0.001** | |  | | |  |  |
| Comorbid Anxiety Diagnosis | | | | | | | | | |  |
| Intercept | -1.69 (-5.43-1.82) | 1.83 | -0.92 | 0.355 | | 74.27(5) | | | 0<0.0001 |  |
| Age | -0.02 (-0.09-0.05) | 0.03 | -0.50 | 0.618 | |  | | |  |  |
| MDD Duration | -0.09 (-0.19-0.00) | 0.05 | -1.81 | 0.071 | |  | | |  |  |
| HRSD_17_ | 0.07 (-0.07-0.22) | 0.07 | 0.94 | 0.347 | |  | | |  |  |
| Comorbid Anxiety Diagnosis | 1.08 (-0.16-2.45) | 0.66 | 1.65 | 0.100 | |  | | |  |  |
| **PCC-ACC/mPFC Connectivity** | **7.31 (3.71-11.83)** | **2.05** | **3.57** | **0.000** | |  | | |  |  |
| Number of Early life Stressors | | | | | | | | | |  |
| Intercept | -0.48 (-4.46-3.43) | 1.99 | -0.24 | 0.808 | | 70.67(5) | | | 0<0.0001 |  |
| Age | -0.03 (-0.1-0.04) | 0.03 | -0.76 | 0.445 | |  | | |  |  |
| MDD Duration | -0.07 (-0.17-0.02) | 0.05 | -1.39 | 0.163 | |  | | |  |  |
| HRSD_17_ | 0.09 (-0.05-0.25) | 0.08 | 1.22 | 0.221 | |  | | |  |  |
| **Number of Early Life Stressors** | **-0.27 (-0.56--0.03)** | **0.13** | **-2.06** | **0.039** | |  | | |  |  |
| **PCC-ACC/mPFC Connectivity** | **6.72 (3.36-10.82)** | **1.88** | **3.57** | **0.000** | |  | | |  |  |
| Body Mass Index |  |  |  |  | |  | | |  |  |
| Intercept | 2.00 (-6.39-2.27) | 2.17 | -0.92 | 0.357 | | 66.73(5) | | | 0<0.0001 |  |
| Age | -0.02 (-0.09-0.05) | 0.03 | -0.55 | 0.582 | |  | | |  |  |
| MDD Duration | -0.09 (-0.19--0.01) | 0.05 | -1.96 | 0.050 | |  | | |  |  |
| HRSD_17_ | 0.08 (-0.06-0.24) | 0.08 | 1.08 | 0.280 | |  | | |  |  |
| Body Mass Index | 0.04 (-0.07-0.16) | 0.06 | 0.75 | 0.455 | |  | | |  |  |
| **PCC-ACC/mPFC Connectivity** | **6.07 (2.63-10.28)** | **1.92** | **3.16** | **0.002** | |  | | |  |  |
| Attention |  |  |  |  | |  | | |  |  |
| Intercept | -1.22 (-5.06-2.40) | 1.88 | -0.65 | 0.515 | | 67.13(5) | | | 0<0.0001 |  |
| Age | -0.02 (-0.09-0.05) | 0.03 | -0.50 | 0.620 | |  | | |  |  |
| MDD Duration | -0.09 (-0.19-0.00) | 0.05 | -1.91 | 0.057 | |  | | |  |  |
| HRSD_17_ | 0.09 (-0.06-0.25) | 0.08 | 1.12 | 0.261 | |  | | |  |  |
| Attention | 0.44 (-0.49-1.57) | 0.52 | 0.83 | 0.406 | |  | | |  |  |
| **PCC-ACC/mPFC Connectivity** | **6.41 (2.97-10.63)** | **1.93** | **3.33** | **0.001** | |  | | |  |  |
| Cognitive Flexibility |  |  |  |  | |  | | |  |  |
| Intercept | -0.97 (-4.87-2.84) | 1.93 | -0.50 | 0.614 | | 66.12(5) | | | 0<0.0001 |  |
| Age | 0.00 (-0.08-0.07) | 0.04 | -0.13 | 0.897 | |  | | |  |  |
| MDD Duration | -0.10 (-0.21--0.01) | 0.05 | -2.07 | 0.038 | |  | | |  |  |
| HRSD_17_ | 0.07 (-0.08-0.23) | 0.08 | 0.88 | 0.381 | |  | | |  |  |
| Cognitive Flexibility | -0.45 (-1.36-0.19) | 0.38 | -1.18 | 0.237 | |  | | |  |  |
| **PCC-ACC/mPFC Connectivity** | **6.41 (2.94-10.69)** | **1.95** | **3.29** | **0.001** | |  | | |  |  |

*Abbreviations:* B = Unstandardized beta coefficient; SE = Standard Error; MDD = Major Depressive Disorder; HRSD_17_ = 17-item Hamilton Rating Scale for Depression;

**Table S2. ACC-mPFC Connectivity Predicting HRSD_17_ Remission with additional Demographic and Clinical variables**

| **Model Predictors** | **B (95% CI)** | **SE** | **Wald** | **p-value** | | **Model Fit** | | | |  |
| --- | --- | --- | --- | --- | --- | --- | --- | --- | --- | --- |
|  |  |  |  |  | | **χ^2^(df)** | | ***p*-value** | |  |
| Decision Speed |  |  |  |  |  | |  | | | |
| Intercept | -1.05 (-4.80-2.57) | 1.85 | -0.57 | 0.572 | | 67.85(5) | | | 0<0.0001 |  |
| Age | -0.02 (-0.09-0.05) | 0.03 | -0.55 | 0.583 | |  | | |  |  |
| MDD Duration | -0.09 (-0.19-0.00) | 0.05 | -1.89 | 0.059 | |  | | |  |  |
| HRSD_17_ | 0.08 (-0.07-0.24) | 0.08 | 1.06 | 0.291 | |  | | |  |  |
| Decision Speed | 0.06 (-0.41-0.80) | 0.27 | 0.21 | 0.831 | |  | | |  |  |
| **PCC-ACC/mPFC Connectivity** | **6.45 (2.99-10.68)** | **1.94** | **3.33** | **0.001** | |  | | |  |  |
| Executive Functioning | | | | | | | | | |  |
| Intercept | -0.93 (-4.71-2.72) | 1.87 | -0.50 | 0.618 | | 67.24(5) | | | 0<0.0001 |  |
| Age | -0.02 (-0.09-0.05) | 0.03 | -0.51 | 0.608 | |  | | |  |  |
| MDD Duration | -0.09 (-0.19-0.00) | 0.05 | -1.88 | 0.060 | |  | | |  |  |
| HRSD_17_ | 0.07 (-0.07-0.23) | 0.08 | 0.97 | 0.332 | |  | | |  |  |
| Executive Functioning | -0.27 (-1.09-0.31) | 0.36 | -0.75 | 0.455 | |  | | |  |  |
| **PCC-ACC/mPFC Connectivity** | **6.38 (2.91-10.65)** | **1.95** | **3.28** | **0.001** | |  | | |  |  |
| Information Processing Speed | | | | | | | | | |  |
| Intercept | -0.93 (-4.75-2.76) | 1.89 | -0.49 | 0.623 | | 65.91(5) | | | 0<0.0001 |  |
| Age | -0.02 (-0.10-0.05) | 0.04 | -0.47 | 0.638 | |  | | |  |  |
| MDD Duration | -0.09 (-0.21-0.00) | 0.05 | -1.77 | 0.077 | |  | | |  |  |
| HRSD_17_ | 0.08 (-0.08-0.24) | 0.08 | 0.95 | 0.341 | |  | | |  |  |
| **Information Processing Speed** | -0.60 (-1.61-0.33) | 0.49 | -1.23 | 0.217 | |  | | |  |  |
| **PCC-ACC/mPFC Connectivity** | **6.78 (3.14-11.29)** | **2.05** | **3.3** | **0.001** | |  | | |  |  |
| Motor Coordination | | | | | | | | | | |
| Intercept | -0.96 (-4.69-2.61) | 1.84 | -0.52 | 0.601 | | 67.78(5) | | | 0<0.0001 |  |
| Age | -0.02 (-0.09-0.04) | 0.03 | -0.63 | 0.530 | |  | | |  |  |
| MDD Duration | -0.09 (-0.19-0.00) | 0.05 | -1.84 | 0.065 | |  | | |  |  |
| HRSD_17_ | 0.08 (-0.07-0.24) | 0.08 | 1.03 | 0.302 | |  | | |  |  |
| Motor Coordination? | -0.26 (-1.82-1.25) | 0.77 | -0.33 | 0.738 | |  | | |  |  |
| **PCC-ACC/mPFC Connectivity** | **6.46 (3.01-10.69)** | **1.93** | **3.34** | **0.001** | |  | | |  |  |
| Response Inhibition |  |  |  |  | |  | | |  |  |
| Intercept | -1.18 (-4.93-2.41) | 1.85 | -0.64 | 0.521 | | 66.89(5) | | | 0<0.0001 |  |
| Age | -0.02 (-0.09-0.05) | 0.03 | -0.52 | 0.605 | |  | | |  |  |
| MDD Duration | -0.08 (-0.18-0.01) | 0.05 | -1.60 | 0.110 | |  | | |  |  |
| HRSD_17_ | 0.08 (-0.07-0.24) | 0.08 | 1.06 | 0.291 | |  | | |  |  |
| Response Inhibition | 0.24 (-0.71-1.36) | 0.51 | 0.48 | 0.633 | |  | | |  |  |
| **PCC-ACC/mPFC Connectivity** | **6.56 (3.13-10.77)** | **1.92** | **3.41** | **0.001** | |  | | |  |  |
| Verbal Memory |  |  |  |  | |  | | |  |  |
| Intercept | -0.76 (-4.59-2.95) | 1.90 | -0.40 | 0.688 | | 66.53(5) | | | 0<0.0001 |  |
| Age | -0.02 (-0.09-0.04) | 0.03 | -0.59 | 0.552 | |  | | |  |  |
| MDD Duration | -0.09 (-0.19-0.00) | 0.05 | -1.88 | 0.060 | |  | | |  |  |
| HRSD_17_ | 0.07 (-0.08-0.23) | 0.08 | 0.90 | 0.366 | |  | | |  |  |
| Verbal Memory | 0.47 (-0.31-1.33) | 0.41 | 1.15 | 0.249 | |  | | |  |  |
| **PCC-ACC/mPFC Connectivity** | **6.59 (3.09-10.90)** | **1.96** | **3.36** | **0.001** | |  | | |  |  |

*Abbreviations:* B = Unstandardized beta coefficient; SE = Standard Error; MDD = Major Depressive Disorder; HRSD_17_ = 17-item Hamilton Rating Scale for Depression;

**Table S2. ACC-mPFC Connectivity Predicting HRSD_17_ Remission with additional Demographic and Clinical variables**

| **Model Predictors** | **B (95% CI)** | **SE** | **Wald** | **p-value** | **Model Fit** | | |  |
| --- | --- | --- | --- | --- | --- | --- | --- | --- |
|  |  |  |  |  | **χ^2^(df)** | ***p*-value** | |  |
| Working Memory | | | | | | | | |
| Intercept | -0.79 (-4.57-2.88) | 1.87 | -0.42 | 0.674 | 67.53(5) | | 0<0.0001 |  |
| Age | -0.02 (-0.09-0.04) | 0.03 | -0.64 | 0.524 |  | |  |  |
| MDD Duration | -0.09 (-0.2--0.01) | 0.05 | -1.92 | 0.055 |  | |  |  |
| HRSD_17_ | 0.08 (-0.07-0.23) | 0.08 | 1.00 | 0.317 |  | |  |  |
| Working Memory | 0.19 (-0.43-0.83) | 0.32 | 0.61 | 0.545 |  | |  |  |
| **PCC-ACC/mPFC Connectivity** | 6.42 (2.94-10.66) | 1.94 | 3.30 | 0.001 |  | |  |  |

*Abbreviations:* B = Unstandardized beta coefficient; SE = Standard Error; MDD = Major Depressive Disorder; HRSD_17_ = 17-item Hamilton Rating Scale for Depression;
