# Supplementary material for: Intrinsic functional connectivity predicts remission on antidepressants: a randomized controlled trial to identify clinically applicable imaging biomarkers
Source: Transl Psychiatry. 2018 Mar 6;8:57. doi: 10.1038/s41398-018-0100-3 (PMC5838245; doi:10.1038/s41398-018-0100-3)
Supplement: Supplementary file 5 — Supplemental Figure Legends [file 41398_2018_100_MOESM5_ESM.docx]

**SUPPLEMENTARY FIGURE LEGENDS**

**Figure S1:** CONSORT chart of the iSPOT-D sample.

**Figure S2:** Whole-brain, voxel-wise results demonstrating: (a) Group differences in baseline functional connectivity with the posterior cingulate cortex seed between remitters and non-remitters. (b) Linear relationship between baseline functional connectivity with the posterior cingulate cortex seed and the percent reduction in Hamilton Depression Rating Scale (HRSD_17_) symptoms. Results for (a) and (b) are displayed at the uncorrected p < 0.001 with a cluster threshold of k > 10 voxels. (c) The overlap between binary and linear associations of treatment outcomes at the FWE-cluster corrected p<0.001 threshold.
